# Cryoglobulinemic Vasculitis: Clinical Analysis of a Case and Its Diagnostic Relevance

**DOI:** 10.7759/cureus.112100

**Published:** 2026-07-05

**Authors:** Valeria Janet Martínez Evangelista, Paola V Rosales Verduzco, Ricardo Cid-Puente, Ana Laura Rodríguez Hernández, Nancy Arreola Sevilla

**Affiliations:** 1 Internal Medicine, Hospital Regional Dr. Valentín Gómez Farías, ISSSTE (Instituto de Seguridad y Servicios Sociales de los Trabajadores del Estado), Jalisco, MEX; 2 Immunology, Biological Sciences School, Universidad Autónoma de Zacatecas, Zacatecas, MEX; 3 Internal Medicine, Hospital Regional General 180 IMSS (Mexican Social Security Institute), Jalisco, MEX

**Keywords:** cryoglobulins, hypocomplementemia, idiopathic, palpable purpura, small-vessel vasculitis

## Abstract

Cryoglobulinemic vasculitis is an immune-complex-mediated small-vessel vasculitis characterized by complement activation and endothelial injury. Its clinical manifestations are heterogeneous and may range from limited cutaneous involvement to systemic disease. We report the case of a 65-year-old woman who developed a symmetrical violaceous dermatosis involving the upper and lower extremities, accompanied by arthralgias and distal paresthesias. Laboratory investigations revealed marked hypocomplementemia and positive circulating cryoglobulins. Histopathological examination of a skin biopsy demonstrated leukocytoclastic vasculitis, leading to the diagnosis of idiopathic cryoglobulinemic vasculitis after exclusion of hepatitis C virus infection, autoimmune diseases, and associated hematologic malignancies, with no evidence of major systemic organ involvement. Treatment with corticosteroid- and cyclophosphamide-based immunosuppression achieved sustained disease control, resulting in approximately 90% symptomatic improvement. This case highlights the importance of recognizing cardinal manifestations, such as palpable purpura, arthralgias, and hypocomplementemia, when evaluating patients with suspected cryoglobulinemic vasculitis, even in the absence of identifiable secondary causes. Early recognition, histopathological confirmation, and a multidisciplinary approach are essential for establishing an accurate diagnosis and implementing timely therapeutic intervention aimed at preventing disease progression and irreversible organ damage.

## Introduction

Cryoglobulinemic vasculitis is an uncommon systemic vasculitis affecting small- and medium-sized blood vessels, mediated by cryoglobulins, which are immunoglobulins that precipitate at temperatures below 37 °C. The deposition of these proteins triggers complement activation, endothelial inflammation, and subsequent vascular injury [1-3]. Clinical manifestations are heterogeneous, ranging from palpable purpura, arthralgia, and fatigue to severe systemic involvement, including glomerulonephritis, peripheral neuropathy, and multiorgan dysfunction, all of which substantially increase morbidity and mortality [4,5].

The diagnosis is based on a combination of clinical and laboratory findings, including the detection of serum cryoglobulins, hypocomplementemia, and positive rheumatoid factor. Identifying the underlying etiology is essential, particularly hepatitis C virus infection, which remains the leading cause of mixed cryoglobulinemia worldwide [2,6]. Treatment should be individualized according to disease severity and may include management of the underlying condition, immunosuppressive therapy with glucocorticoids and rituximab in moderate-to-severe cases, and plasmapheresis for life-threatening manifestations. Despite advances in diagnostic strategies and therapeutic options, idiopathic cryoglobulinemic vasculitis remains a diagnostic challenge because of its rarity, heterogeneous clinical presentation, and the need for systematic exclusion of secondary causes.

This case report aims to highlight the diagnostic challenges associated with idiopathic cryoglobulinemic vasculitis, emphasizing the importance of systematically excluding secondary causes and the value of integrating clinical, laboratory, and histopathological findings within a multidisciplinary framework to achieve timely diagnosis and individualized management.

## Case presentation

A 65-year-old woman, a homemaker with a family history significant for type 2 diabetes mellitus, psoriasis, and pancreatic cancer, presented to our institution. Prior to symptom onset, she was fully independent in all activities of daily living. Her past medical history was notable for an ischemic cerebrovascular event two years earlier, with magnetic resonance imaging demonstrating a lacunar infarction and a modified Rankin Scale score of 1 at follow-up.

The patient sought medical attention because of the progressive development of a symmetrically distributed dermatosis involving both the upper and lower extremities. Dermatologic examination revealed multiple palpable purpuric lesions associated with residual post-inflammatory hyperpigmented macules (Figure 1). She also reported symmetrical inflammatory arthralgia with predominant involvement of the bilateral radiocarpal and patellofemoral joints, together with distal sensory loss affecting the distal third of both lower extremities.

**Figure 1:**
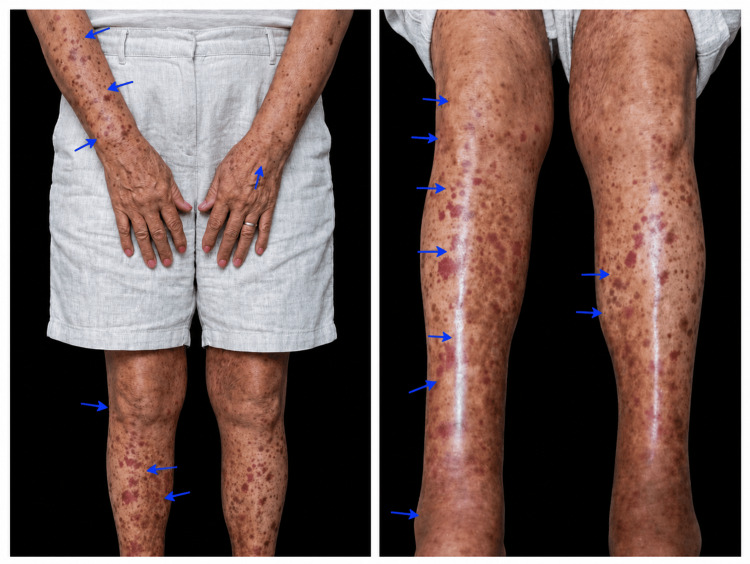
Symmetrically distributed palpable purpuric lesions affecting the upper and lower extremities, identified by the blue arrows

A comprehensive diagnostic evaluation for vascular purpura was undertaken to systematically exclude infectious, autoimmune, and lymphoproliferative disorders, as well as coagulation abnormalities. Serologic testing for hepatitis C virus and human immunodeficiency virus was non-reactive, while an extensive autoimmune evaluation, including antinuclear antibodies (ANA), anti-double-stranded DNA antibodies (anti-dsDNA), extractable nuclear antigen antibodies (ENA), and antineutrophil cytoplasmic antibodies (ANCA), yielded negative results. As part of the evaluation for an underlying monoclonal gammopathy, serum protein electrophoresis was performed as an initial screening test and demonstrated no evidence of a monoclonal protein. Rheumatoid factor was markedly elevated (205.4 IU/mL). Additional laboratory abnormalities included lymphopenia (900 cells/µL), reduced complement C3 (0.84 g/L), and marked C4 hypocomplementemia (0.02 g/L), as summarized in Table 1. Although an extended complement profile was not available, the combination of reduced C3 and profoundly decreased C4 strongly supported an immune complex-mediated vasculitic process.

**Table 1:** Laboratory findings during hospitalization % = percentage; IU/mL = international units per milliliter; U/mL = assay units per milliliter; g/L = grams per liter; mg/dL = milligrams per deciliter; mm/h = millimeters per hour; µL = microliter; RF = rheumatoid factor; ANA = antinuclear antibodies; anti-dsDNA = anti-double-stranded DNA antibodies; anti-CCP = anti-cyclic citrullinated peptide antibodies; ACPA = anti-citrullinated protein antibodies; CRP = C-reactive protein; ESR = erythrocyte sedimentation rate; HIV = human immunodeficiency virus; HCV = hepatitis C virus

Test	Patient Value	Laboratory Reference Range
Cryoglobulins	Present (>5%)	Negative (0%)
Rheumatoid Factor (RF)	205.4 IU/mL	<14 IU/mL
Antinuclear Antibodies (ANA)	1:80, dense fine speckled nuclear pattern	Negative or <1:80
Anti-double-stranded DNA antibodies (anti-dsDNA)	Negative	Negative (<25–30 IU/mL)
Anti-cyclic Citrullinated Peptide Antibodies (Anti-CCP, ACPA)	Negative	Negative (<20 U/mL)
Complement C3	0.84 g/L	0.9–1.8 g/L
Complement C4	0.02 g/L	0.10–0.40 g/L
C-reactive Protein (CRP)	10 mg/dL	<0.5 mg/dL
Erythrocyte Sedimentation Rate (ESR)	53 mm/h	0–15 mm/h
Immunoglobulin G (IgG)	16 g/L	7–16 g/L
Immunoglobulin M (IgM)	2.9 g/L	0.4–2.3 g/L
Immunoglobulin A (IgA)	3 g/L	1–4 g/L
White Blood Cell Count	4,200/µL	4,000–10,000/µL
Neutrophil Count	5,500/µL	1,500–7,500/µL
Lymphocyte Count	900/µL	1,000–4,000/µL
Human Immunodeficiency Virus (HIV)	Non-reactive	Non-reactive
Hepatitis C Virus (HCV)	Non-reactive	Non-reactive

The diagnostic approach was guided by two principal clinical manifestations: palpable purpura and inflammatory arthralgias. Neurological examination revealed no focal neurological deficits, preserved muscle strength (Medical Research Council grade 5/5 in all extremities), and normal deep tendon reflexes. Sensory examination, performed by assessment of light-touch sensation, demonstrated decreased tactile perception over the distal third of both lower extremities without objective motor deficits or additional neurological abnormalities, consistent with mild distal peripheral sensory involvement.

A skin biopsy was subsequently obtained from an active purpuric lesion. Histopathological examination demonstrated leukocytoclastic vasculitis (Figure 2). Cryoglobulin testing was subsequently performed at a specialized reference laboratory following standardized pre-analytical and analytical procedures. Blood samples were collected, transported, and maintained at 37 °C until serum separation to prevent premature cryoprecipitation. Qualitative analysis demonstrated elevated circulating cryoglobulins (>5%), thereby confirming cryoglobulinemia. However, cryoglobulin typing was not available; therefore, differentiation among type I, type II, and type III cryoglobulinemia could not be established. Such classification requires additional immunochemical characterization of the cryoprecipitate, most commonly by immunofixation electrophoresis, which was not available at our institution. Nevertheless, the combination of positive circulating cryoglobulins, markedly reduced C4 levels, elevated rheumatoid factor, compatible histopathological findings, and the systematic exclusion of infectious, autoimmune, and lymphoproliferative disorders provided sufficient diagnostic evidence to establish the diagnosis of idiopathic cryoglobulinemic vasculitis.

**Figure 2:**
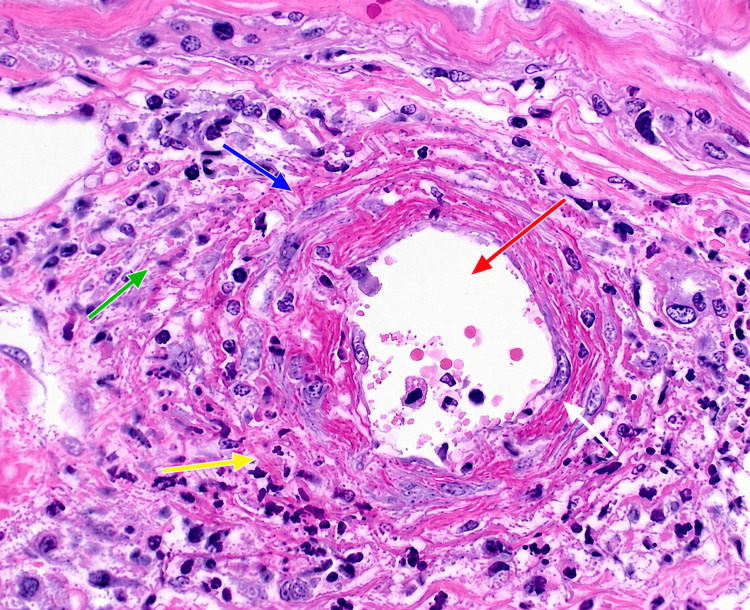
Hematoxylin and eosin-stained skin biopsy, high-power field (original magnification ×400), demonstrating a small dermal blood vessel with fibrinoid necrosis (blue arrow), prominent neutrophilic infiltration (green arrow), leukocytoclasia (yellow arrow), erythrocyte extravasation (red arrow), and endothelial damage (white arrow), findings characteristic of leukocytoclastic vasculitis.

Throughout the diagnostic evaluation, no clinical or laboratory evidence of major systemic organ involvement was identified, including renal, pulmonary, or gastrointestinal disease.

Initial treatment consisted of intravenous methylprednisolone (500 mg/day) administered for three consecutive days, followed by a gradual oral prednisone taper. Because active cutaneous vasculitis persisted despite high-dose glucocorticoid therapy and rituximab was unavailable at our institution, intravenous cyclophosphamide was selected as induction therapy as an evidence-based alternative in accordance with current recommendations for patients in whom rituximab is not available. Six induction pulses were administered at a dose of 0.5 g/m² per cycle, resulting in marked improvement of the cutaneous lesions and inflammatory arthralgias.

Following successful induction therapy, maintenance immunosuppression was individualized according to the patient's clinical response and progressive corticosteroid tapering. Methotrexate was initially introduced as a steroid-sparing agent but was subsequently replaced with mycophenolate mofetil as part of the long-term maintenance strategy adopted by the treating rheumatology team. Tacrolimus was later incorporated into an individualized maintenance regimen based on the patient's sustained clinical response and ongoing therapeutic requirements. Folic acid was administered concomitantly during methotrexate therapy to minimize treatment-related toxicity, and celecoxib was prescribed for symptomatic relief of residual inflammatory arthralgias. At the six-month follow-up, the patient remained clinically stable, with approximately 90% improvement in cutaneous manifestations, arthralgias, and sensory symptoms, without evidence of disease relapse, new systemic organ involvement, or treatment-related adverse events.

## Discussion

Cryoglobulinemic vasculitis represents a clinical challenge that requires a high index of suspicion in the presence of Meltzer’s triad, which is fully manifested in fewer than 30% of cases [6]. A thorough assessment of the cardinal signs and symptoms of the disease becomes the cornerstone of an accurate diagnosis, as it allows clinicians to establish the appropriate diagnostic pathway and therapeutic approach. Although the identification of idiopathic cryoglobulinemic vasculitis (CV) remains challenging because of its low prevalence, a diagnosis based on the integration of clinical, laboratory, and histopathological findings has a substantial impact on patient prognosis. In this regard, it is imperative to conduct a comprehensive evaluation to exclude secondary causes, particularly hepatitis C virus infection, autoimmune diseases, and malignancies [2,6], reserving the term “idiopathic” exclusively for cases in which no clear underlying trigger can be identified [7], as documented in the present case.

During the diagnostic workup, hypocomplementemia and lymphopenia acted as pivotal immunological markers; specifically, consumption of the C4 complement fraction has been highlighted in the literature as a highly sensitive indicator of this condition [8]. Likewise, the histopathological finding of leukocytoclastic vasculitis on skin biopsy was crucial in confirming immune complex-mediated vascular injury [9]. Given the initial refractoriness to glucocorticoid therapy, escalation to intravenous cyclophosphamide pulse therapy proved instrumental in halting disease progression, supporting the role of this agent as an effective therapeutic alternative in patients with moderate-to-severe disease manifestations [10]. Finally, the therapeutic success observed, with approximately 90% clinical improvement, underscores the effectiveness of a multimodal and interdisciplinary maintenance strategy in optimizing functional outcomes and preventing irreversible organ damage [6,10].

The differential diagnostic approach following the histopathological identification of leukocytoclastic vasculitis required the exclusion of other small-vessel vasculitides through the assessment of disease-specific biomarkers and key clinical manifestations [11]. ANCA-associated vasculitides were ruled out based on negative serologic testing [12]; IgA vasculitis was excluded because of normal serum IgA levels and the absence of gastrointestinal manifestations [13]; and hypersensitivity vasculitis was considered unlikely given the profound hypocomplementemia and concomitant peripheral neurological involvement observed in this patient [14]. The defining criteria supporting the diagnosis of cryoglobulinemic vasculitis included the clinical triad of palpable purpura, arthralgias, and sensory neuropathy, in strict concordance with positive serum cryoglobulin testing [1]. The condition was ultimately classified as idiopathic following an exhaustive evaluation that excluded hepatitis C virus infection [6], connective tissue diseases, and hematologic malignancies, while demonstrating preserved renal function and the absence of major systemic organ involvement [15].

## Conclusions

Idiopathic cryoglobulinemic vasculitis remains a diagnostic challenge because of its heterogeneous clinical presentation and the need to systematically exclude secondary causes, particularly hepatitis C virus infection, connective tissue diseases, and lymphoproliferative disorders. This case highlights the importance of integrating clinical, laboratory, and histopathological findings, emphasizing the diagnostic value of palpable purpura, persistent hypocomplementemia, circulating cryoglobulins, and histopathological confirmation of leukocytoclastic vasculitis, particularly in patients presenting with an incomplete form of Meltzer’s triad. Furthermore, this report illustrates the potential value of early disease recognition and close multidisciplinary collaboration among Internal Medicine, Dermatology, and Rheumatology specialists in facilitating timely diagnosis, guiding individualized therapeutic decisions, and supporting favorable clinical outcomes.

## References

[1] Ferri C (2008). Mixed cryoglobulinemia. Orphanet J Rare Dis.

[2] Roccatello D, Saadoun D, Ramos-Casals M (2018). Cryoglobulinaemia. Nat Rev Dis Primers.

[3] Túlio M, Carvalho L, Bana e Costa T, Chagas C (2017). Mixed cryoglobulinemia: a diagnostic and therapeutic challenge. BMJ Case Rep.

[4] Demirkesen C (2017). Approach to cutaneous vasculitides with special emphasis on small vessel vasculitis: histopathology and direct immunofluorescence. Curr Opin Rheumatol.

[5] Sise ME, Bloom AK, Wisocky J (2016). Treatment of hepatitis C virus-associated mixed cryoglobulinemia with direct-acting antiviral agents. Hepatology.

[6] Muchtar E, Magen H, Gertz MA (2017). How I treat cryoglobulinemia. Blood.

[7] De Vita S, Soldano F, Isola M (2011). Preliminary classification criteria for the cryoglobulinaemic vasculitis. Ann Rheum Dis.

[8] Zignego AL, Gragnani L, Visentini M (2026). Cryoglobulinemia: an update on classification, pathophysiology, clinical presentation, and management. J Intern Med.

[9] Carlson JA (2010). The histological assessment of cutaneous vasculitis. Histopathology.

[10] Núñez-Conde A, de la Hera Fernández J, García-Méndez S (2023). Nonviral cryoglobulinemic vasculitis: an updated review for clinical practice. Vessel Plus.

[11] Jennette JC, Falk RJ, Bacon PA (2013). 2012 revised International Chapel Hill consensus conference nomenclature of vasculitides. Arthritis Rheum.

[12] Geetha D, Jefferson JA (2020). ANCA-associated vasculitis: core curriculum 2020. Am J Kidney Dis.

[13] Audemard-Verger A, Pillebout E, Guillevin L, Thervet E, Terrier B (2015). IgA vasculitis (Henoch-Shönlein purpura) in adults: diagnostic and therapeutic aspects. Autoimmun Rev.

[14] Ortiz-Sanjuán F, Blanco R, Hernández JL (2014). Drug-induced cutaneous vasculitis: a study of 466 patients. Medicine (Baltimore).

[15] Terrier B, Karras A, Kahn JE (2013). The spectrum of type I cryoglobulinemia vasculitis: new insights based on 64 cases. Medicine (Baltimore).

